# Treatment as prevention within the framework of current guidelines

**DOI:** 10.1186/1758-2652-13-S4-O12

**Published:** 2010-11-08

**Authors:** J Montaner

**Affiliations:** 1 BC Centre for Excellence in HIV/AIDS, Vancouver, British Columbia, Canada

## 

While an outright cure or a preventive vaccine for HIV/AIDS remain elusive, remarkable advances in HIV treatment have been achieved over the past two decades. Most significant among these advances is the development of highly active antiretroviral therapy (HAART).

Available evidence increasingly supports the notion that the viral load suppression achieved by HAART has a preventive impact on the transmission of HIV. Hence, expanding HAART coverage to all those in medical need, represents a key strategy to not only decrease HIV and AIDS related morbidity and mortality but also to dramatically reduce HIV transmission by all routes.

In British Columbia, the expansion of HAART coverage has been associated with a substantial reduction in HIV new diagnoses (Montaner et al, Lancet, August 18th 2010). The role of treatment as prevention has now been formally incorporated within the UNAIDS global AIDS control strategy, under the "Treatment 2.0" initiative.

